# Fast and Accurate 3D Measurement Based on Light-Field Camera and Deep Learning

**DOI:** 10.3390/s19204399

**Published:** 2019-10-11

**Authors:** Haoxin Ma, Zhiwen Qian, Tingting Mu, Shengxian Shi

**Affiliations:** 1 School of Mechanical Engineering, Shanghai Jiao Tong University, 800 Dongchuan Rd., Shanghai 200240, China; marquess@sjtu.edu.cn; 2 VOMMA (Shanghai) Technology Co., Ltd, Shanghai 200240, China; zwqian@vommatec.com; 3 School of Computer Science, University of Manchester, Kilburn Building, Manchester M13 9PL, UK; tingting.mu@manchester.ac.uk

**Keywords:** light-field imaging, depth estimation, texture-less and reflective areas

## Abstract

The precise combination of image sensor and micro-lens array enables light-field cameras to record both angular and spatial information of incoming light, therefore, one can calculate disparity and depth from one single light-field image captured by one single light-field camera. In turn, 3D models of the recorded objects can be recovered, which means a 3D measurement system can be built using a light-field camera. However, reflective and texture-less areas in light-field images have complicated conditions, making it hard to correctly calculate disparity with existing algorithms. To tackle this problem, we introduce a novel end-to-end network VommaNet to retrieve multi-scale features from reflective and texture-less regions for accurate disparity estimation. Meanwhile, our network has achieved similar or better performance in other regions for both synthetic light-field images and real-world data compared to the state-of-the-art algorithms.

## 1. Introduction

With recent developments in lenslet-based light-field camera technology [1], especially those commercially available products from Lytro [2] and Raytrix [3], depth estimation from light-field images has been a niche topic in computer vision. Based on the two-plane parameterization [4], light-field images can be used to generate multi-view images with slightly different view points and refocused images with different focal planes [5]. With these advantages, various algorithms [6,7,8] have been developed to estimate depth information from single light-field image. Such depth information, when combined with sophisticated metric calibration techniques [9,10], could generate very dense point clouds as well as corresponding textures. This could be very attractive to 3D modeling and 3D geometry measurement community, especially for outdoor applications.

To further improve depth estimation accuracy for light-field images, challenges induced by small viewing angle of lenslet-based light-field camera need to be properly addressed. A series of algorithms have, therefore, been proposed to solve the occlusions [8,11,12], narrow baseline [6], and intrinsic component recovering [13] difficulties. Although computationally expensive [14], these algorithms have been successfully applied in high-texture, non-reflective, and Lambertian surfaces. However, depth estimation from reflective and texture-less light-field images remain a challenge for most of current algorithms because points in these regions have the same RGB values, making it hard to find the correct corresponding points. Attempts have been made to recover depth information for these regions with the help of shape-from-shading [15,16,17], but doing so would need prior knowledge of illumination (captured or estimated), and is generally limited to Lambertian surfaces or surfaces with uniform reflectance [18]. As pointed out by Zhu et al. [19], depth estimation from reflective and texture-less light-field images has not yet been fully studied and more attentions are needed before the light-field imaging could become an attractive alternative for 3D modeling and 3D measurement community.

In this paper, we construct a fast and accurate 3D measurement system based on a single light-field camera and propose a new end-to-end network that specifically addresses the problem of light-field disparity estimation in reflective and texture-less areas by enlarging the receptive field in early layers of the network so that it will be able to infer the accurate depth value of these regions from the value of their edges, while maintains a similar or better performance in other regions compared to existing algorithms. For that purpose, we use a high-resolution industry grade light-field camera and our proposed network takes all of sub-aperture images (SAIs) directly as inputs to make full use of information recorded by light-field cameras.

Our paper is organized as follows. In Section 2, we introduce previous works in the fields of light-field, depth estimation, and neural network; in Section 3, we explain our measurement system and neural network design in details; in Section 4, we perform various experiments with real world light-field images captured by a Lytro Illum camera, and compare our results with those of state-of-the-art algorithms, also, we measure a series of standard gauge blocks with our proposed system to validate its accuracy; in Section 5, we conclude our research and point out future work and possible improvements.

## 2. Related Work

The concept of light-field has a pretty long history. Adelson and Bergen [20] parameterized light rays with the plenoptic function L(x,y,z,θ,φ), where (x,y,z) stands for its 3D coordinate in space and (θ,φ) stands for its angle of propagation. By assuming constant irradiance of a light ray along its path of propagation, later researchers [21,22] reduced this to a 4D function which can be denoted as L(x,y,u,v) where (x,y) and (u,v) are the coordinates of points where the light ray intersects with two parallel planes. Based on these advances, Ng et al. [1] proposed a micro-lens array (MLA) based light-field camera where a high-resolution MLA was installed between the main lens and the camera sensor to record the plenoptic function of incoming light rays. Taking advantage of such light-field cameras, researchers have proven the feasibility of applying single light-field camera for 3D object reconstruction tasks in volumetric flow measurement and 3D geometry reconstruction [23,24,25,26].

Light-field images can be re-sampled to SAIs with epipolar constraint, which can be processed in a similar fashion as stereo matching [6]. Therefore, depth from light-field can be estimated based on correspondence. Jeon et al. [6] proposed a correspondence method based on phase shift theorem to solve the narrow baseline problem, and improve the algorithm by using a cascade random forest to predict accurate depth value from matching costs [7]. However, as Hane et al. [27] has demonstrated, correspondence based methods will not lead to a confident depth estimation in reflective and texture-less area, as many different disparities lead to low matching costs.

On the other hand, the light-field is commonly represented as multi-orientation epipolar plane images(EPIs) [28]. Each of the lines on EPIs corresponds to the projection of a 3D point in space, and the various slopes can be represented as disparity, from which depth can be deducted. Based on the rich structure of EPIs, depth can be analyzed for more complex scenes, such as occlusion areas [29,30]. Johannsen et al. [30] used sparse coding on patches of the EPI to find those dictionary elements which best describe the patch. Zhang et al. [8] proposed an EPI-based Spinning Parallelogram Operator(SPO), which estimates the orientation of epipolar lines and is robust to occlusions. And Sheng et al. [11] improved the method to achieve better accuracy by using multi-orientation EPIs. Schilling [12] proposed a local optimization scheme based on the PatchMatch algorithm, which not only improved object boundaries, but also smooth surface reconstruction.

**Figure 1:**
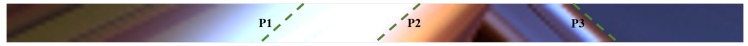
EPI for reflective (between P1 and P2) and texture-less (left from P3) regions. It’s clear that all pixels in these regions have the same RGB value.

Furthermore, various recently proposed EPI-based neural networks [13,31,32,33,34] have shown promising performance in light-field depth estimation. Heber et al. [32] used Convolutional Neural Networks (CNN) to predict EPI line orientations, and then developed an end-to-end deep network architecture to predict depth [33]. Alperovich et al. [13] present a fully convolutional autoencoder for light-field images, which can be decoded in a variety of ways to acquire disparity map, diffuse, and specular intrinsic components. Feng et al. [34] proposed FaceLFnet based on dense block and EPIs from horizontal and vertical SAIs. Shin and Jeon [31] introduced a deep learning-based approach EPINET for light-field depth estimation that achieves accurate results and fast speed. However, since EPI slopes are calculated primarily from neighboring pixel values, as demonstrated in Figure 1, EPI slopes cannot be correctly calculated for reflective and texture-less regions because all pixels in these areas have the same value.

The aforementioned algorithms are only feasible in ordinary non-reflective high-texture regions. For mirror-like reflective or low-texture surfaces, Wanner and Goldluecke [29,35] estimated the slope of epipolar lines by using the second order structure tensor to allow the reconstruction of multi-layered depth maps. They succeeded in accurately estimating depth for mirror-like surfaces and transparent objects. Tao et al. [36] combined the correspondence, defocus cue, and the shape of shading method to refine depth estimation results for Lambertian surfaces. Their method acquired accurate depth for surface of a model shell, a gloss and low-texture surface. Johannsen et al. [30] proposed sparse light-field coding to decompose the light-field of specular surfaces into different superimposed layers, which can leverage the depth estimation for these regions.

Neural networks, especially CNN, have demonstrated their advantages over traditional methods in numerous research fields, and researchers in the field of neural network have carried out more and more works focusing on network structure and learning techniques. Chen et al. [37] proposed atrous convolution which can enlarge the field-of-view, in other words, the receptive field of neural networks, without increasing the number of parameters or the amount of computation, and demonstrated its effectiveness in semantic segmentation. Also, depthwise separable convolution [38,39] has been proposed to greatly decrease the parameter number while maintain a similar performance. And it has shown its feasibility in various fields such as image classification [38]. Moreover, novel techniques of batch normalization [40] and residual neural networks [41] have accelerated the training of deep neural networks while keeping them robust. Efficiency of those methods have been successfully verified by corresponding authors in the field of image classification. We are inspired by these advances and seek to take advantage of them to address the problem of accurate depth estimation for reflective and texture-less areas.

## 3. Our Method

### 3.1. Measurement System Configuration

**Figure 2:**
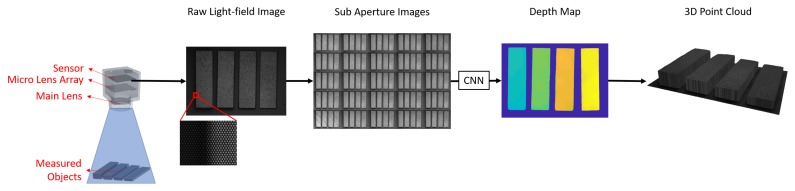
Our proposed measurement system.

As shown in Figure 2, the proposed measurement system consists of a light-field camera, a main lens ( Nikon AF Micro-Nikkor 200 mm f/4D IF-ED, Tokyo, Japan), and coaxial light (OPT, LH-90C-24V-R, Dongguan, China). The camera has a pixel resolution of 8000 × 5500 and MLA resolution of 1200 × 900. The camera is calibrated following the procedures introduced in [26]. In the measurement process, the system first captures raw light-field images of the measured object, which are the collection of individual images beneath each micro-lens of the whole MLA, and then generates multi-perspective SAIs from raw light-field images by taking the same pixel beneath each micro-lens and combining them in the same order as that of the micro-lens they are from [5].

### 3.2. Depth Estimation Neural Network

#### 3.2.1. Network Design

After rendering SAIs from raw light-field image, the task at hand is to estimate depth map (disparity) with our proposed neural network. As demonstrated above, depth for reflective and texture-less areas cannot be accurately estimated if we only examine these areas locally. However, if we take a step back and examine a larger region, we will find that, the disparities for the edges of these areas can be easily calculated. Also, since the disparity values inside an object should be continuous, we can let the network estimate the disparities of reflective and texture-less areas based on those of their edges. Therefore, if we can enable the network to “step back” and “see” a larger region and combine this information with local features extracted from smaller regions, there should be performance improvement for texture-less and reflective areas.

In other words, our network should be able to extract multi-scale features from light-field images. However, this means we need to perform multi-scale convolutions, which may lead to heavy computational burden. Therefore, we also need to decrease parameter number when designing our network.

**Figure 3:**
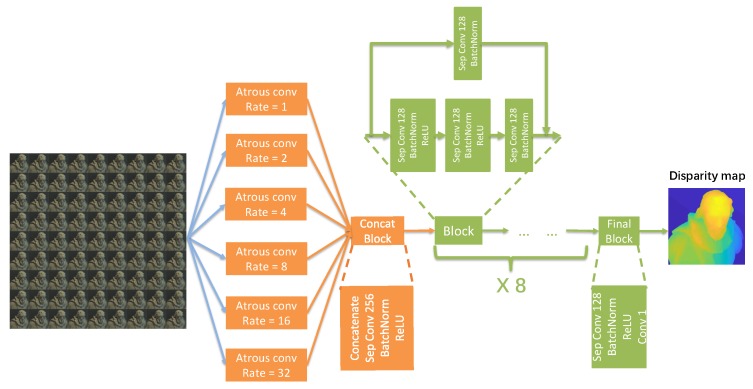
The structure of our network.

In order to let the network extract features from both small and large regions, we concatenate all SAIs in channel axis. Then, we take the concatenated data as input. For example, in Figure 3, we take 9-by-9 SAIs where each SAI has three channels of RGB. We first concatenate the SAIs along channel axis to have a 81 × 3-channel input image. Then, we feed this input to the convolution layers with both small and large receptive field. This way, the network will be able to extract both local and global features directly from SAIs. As stated previously, we aim to enlarge the receptive fields of the network in earlier layers, we design our network as shown in Figure 3. The network consists of two main parts, the feature pyramid whose purpose is to extract multi-scale low-level features from input SAIs, and a series of residual blocks whose purpose is to encode these low-level features into high-level ones and infer pixel-wise disparity values from them.

First, we use a feature pyramid consisted of atrous convolution with increasing dilation rates to extract multi-scale features. From the multi-scale features, our network can infer disparity values for reflective and texture-less areas from their surroundings. The feature pyramid we proposed consists of 6 atrous convolution layers with dilation rates of 1 (no dilation), 2, 4, 8, 16, and 32 separately. We adopt this structure because, as demonstrated by Chen et al. [37], atrous convolution up-samples the convolutional kernels by padding zeros in between trainable parameters, which can effectively enlarge receptive field while keeping a rather low parameter number and computation amount. Therefore, we use atrous convolutions of multiple dilation rates to construct our feature pyramid in order to combine results from both large and small receptive fields in earlier layers to capture both local and global features so that, for reflective and texture-less regions, the network can “take a step back” and view a larger picture. And in turn, the network can learn to infer disparity values for these regions from their neighborhood.

The outputs from different layers of the pyramid, in other words low level multi-scale features, are concatenated along the channel axis and passed to a depthwise separable convolution layer to encode these outputs into higher-level features. After this, we apply a series of residual blocks followed by one single convolution layer to have the final output. One residual block has two passes, one shortcut of a depthwise separable convolution layer, and another pass consisted of three consecutive depthwise separable convolution layers. The last convolution layer has one single 1 × 1 kernel while all other convolutional kernels are of 3 × 3 in size. The outputs from two passes are added together to get the output of this one residual block. By passing the low-level features through a series of residual blocks, we aim to extract high-level information from the multi-scale features captured by the feature pyramid and encode them into the disparity information we need.

We choose to use residual blocks mainly for two reasons. First, our network consists of a large number of layers, which makes it prone to the vanishing gradient problem, and as demonstrated by He et al. [41], residual structure can avoid this problem by re-introducing outputs from shallower layers in the network to compensate for the vanishing data. Second, deeper network means larger number of parameters, which increase computational burden. For that reason, we use depthwise separable convolutions in substitution of normal convolutions to decrease parameter number and speed up training.

All convolution layers in our network is followed by a batch normalization layer and a ReLU activation layer except for the last one.

Take the input in Figure 3 as example again. We feed the input to the network, and make 6 copies of the input, each passing to a atrous convolution layer with different dilation rate. So far, our network will extract multi-scale features from the input. Then, we concatenate the outputs from these 6 layers together, and pass the concatenated features into the residual blocks. This way, the rest part of our network will infer the disparity value with both local and global features taken into account. Therefore, for texture-less and reflective areas, our network will be able to infer the disparity values from their neighborhood.

#### 3.2.2. Loss Function

Since there are shortcuts in the residual blocks, texture of the input images may be preserved in the final output. Therefore, an effective loss function should enforce not only smaller value difference but higher structural similarity between network output and ground truth as well.

Most of the previous studies employ mean absolute error(MAE) between network estimation $d_{i}$ and its ground truth $g_{i}$ as loss function to enforce accuracy for network output:

(1) $$l_{MAE}=\sum_{i=1}^{N}\frac{|D_{i}|}{N}$$

where *N* is the total number of pixels, and $D_{i}=d_{i}-g_{i}$ is the difference between network estimation and its ground truth at the *i*th pixel. However, as illustrated in [42], this loss is insensitive to distortion and blur of edges. Therefore, we employ the following loss to penalize errors around edges more:

(2) $$l_{grad}=\sum_{i=1}^{N}\frac{|\nabla_{x}\left(D_{i}\right)\left|+\right|\nabla_{y}\left(D_{i}\right)|}{N}$$

where $\nabla_{x}$ is spatial gradient in *x*-axis, and $\nabla_{y}$ is that in *y*-axis. To further improve fine details of depth maps, we consider yet another loss from [42], which measures accuracy of the normal to the surface of an estimated depth map with respect to its ground truth:

(3) $$l_{normal}=1-\sum_{i=1}^{N}\frac{\cos<{\vec{n}}_{i}^{d},{\vec{n}}_{i}^{g}>}{N}$$

where ${\vec{n}}_{i}^{d}=\left(-\nabla_{y}d_{i},-\nabla_{x}d_{i},1\right)$, ${\vec{n}}_{i}^{g}=\left(-\nabla_{y}g_{i},-\nabla_{x}g_{i},1\right)$, and $\cos<\vec{a},\vec{b}>$ stands for the cosine value of the angle between two vectors $\vec{a}$ and $\vec{b}$.

Finally, we use weighted sum of the above loss functions to train our network.

$$loss=\lambda_{1}l_{MAE}+\lambda_{2}l_{grad}+\lambda_{3}l_{normal}$$

where $\lambda_{1},\lambda_{2},\lambda_{3}$ are coefficients for different terms.

#### 3.2.3. Training Details

We use data provided by [13] along with the additional data provided by the benchmark [43] as training data. These two datasets both provide light-field images in the form of SAIs and ground truth depth and disparity maps all in the size of 512 × 512 pixels. This form of light-field image and ground truth data is the same as real-world light-field cameras and is compatible with our measurement system introduced in Section 3.1. Since the data amount is not very large, we augment the data by flipping, color inversion, and cropping into mini-batch. To generate training data, we flip the data up-down, left-right, and up-down plus left-right, then invert image color, and finally cut them into mini-batches of size 64. There are totally 116 pairs of light-field image and ground truth before augmentation, and we generate more than 104,400 pairs of training data using this augmentation method. There are also validation dataset containing eight pairs of light-field image and ground truth in the size of 512 × 512 pixels provided by the benchmark and we use that to validate our network.

The learning rate begins from 0.001 and decays every 10 epochs by a factor of 0.5 until it falls below $1\times 10^{-8}$. The training process takes about three days with Intel E5-2603 v4 @1.7 GHz, 64 GB RAM, and Nvidia GeForce GTX 1080Ti. And for loss function, we set all coefficients to 1. In each epoch during the training process, we shuffle the training data and divide all the 104,400 pairs of training data into batches each containing 128 pairs and feed them to the network one batch at a time until every pair has been fed.

## 4. Experiments

### 4.1. Qualitative Evaluation on Depth Estimation Algorithms

**Figure 4:**
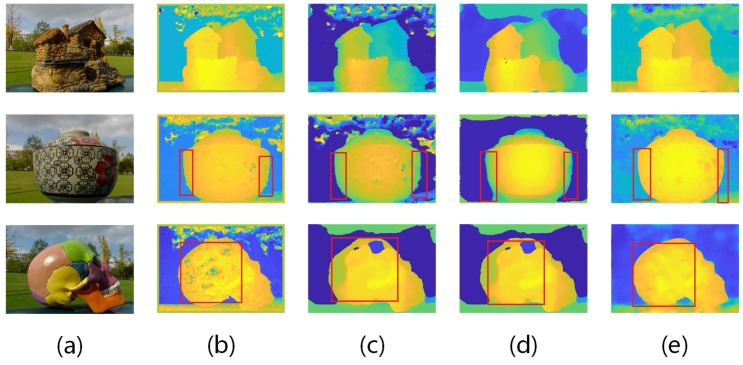
Lytro results. (**a**) thumbnail; (**b**) [31]; (**c**) [8]; (**d**) [6]; (**e**) Ours(VommaNet).

As most of the previous researches test their results with light-field images captured by Lytro cameras, to qualitatively evaluate the performance of our depth estimation algorithm, we perform a number of experiments on real world light-field images captured by a Lytro Illum camera, where we compare results from [6,8,31] with those from our network. Note that for the existing algorithms we comparing with, we directly use the codes and models that the authors published. Notice that to compare with another CNN algorithm [31], we re-train our network from scratch using the same dataset as mentioned in [31]. We capture three different real world scenes where texture-less and reflective areas as well as ordinary ones are present, and process them with different state-of-the-art algorithms as well as our proposed networks. The results are shown in Figure 4. As can be seen from the figure, the first scene is of a model stone house, which is full of texture and not reflective at all; the second scene is of a ceramic bowl, which is pretty rich of texture but quite reflective; the third scene is of a model skull, which is lack of texture, reflective, and even overexposed at some part.

From the first scene of Figure 4, we can see that our network yields better or similar results. We preserve sharp outline of the stone house model and clear detail structures inside the model. Meanwhile, from the second scene we can see that result from our network is better, especially at the edge of the bowl where other algorithms tend to be spiky while ours sharp and clear. Also, from the third scene we can see that for reflective and texture-less areas, e.g., the highlighted rear part of the skull, result from our network remains smooth and accurate while existing algorithms clearly fail, generating absurd values.

### 4.2. Quantitative Evaluation on Benchmark Data

We compared our algorithm with other state-of-the-art algorithms among the benchmark data provided by [43], and the mean squared error values and runtime(reported by corresponding authors) are listed in Table 1. The benchmark provides not only 16 synthetic scenes where both light-field images and ground truth, including depth and dispartiy, are available, but also 12 other synthetic scenes divided into 3 classes. These 12 scenes are used to evaluate the performance of different algorithms. For details about the benchmark, please refer to [43]. The performance of our algorithm and the compared algorithms is listed in Table 1. The data in Table 1 comes from the benchmark directly. The MSE values are calculated by the benchmark based on the outputs that the authors upload to the benchmark. And the runtime values are reported by the authors themselves. Since the benchmark requires authors to upload the outputs of their own algorithms for evaluation, we don’t have the quantitative evaluation for [6] because this algorithm came out before the benchmark and the authors haven’t uploaded their results to the benchmark for evaluation. As can be seen in Table 1, our algorithm achieves the lowest MSE value while maintaining a fast runtime.

### 4.3. 3D Geometry Reconsecration Accuracy Assessment

**Figure 5:**
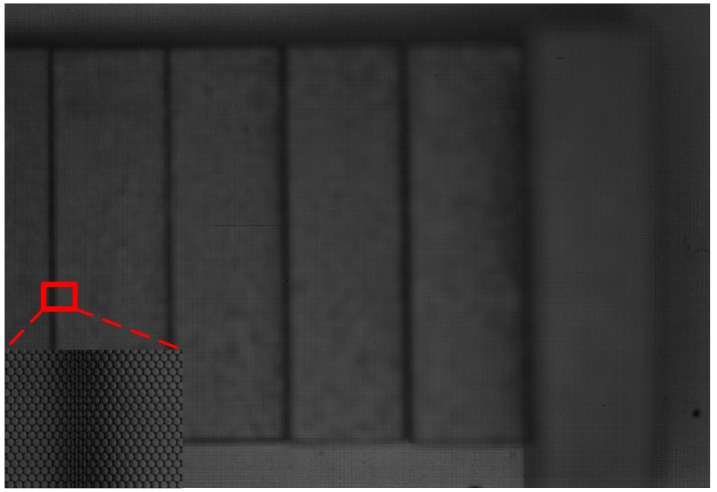
Raw light-field image of standard gauge blocks captured by VOMMA Optec camera.

**Figure 6:**
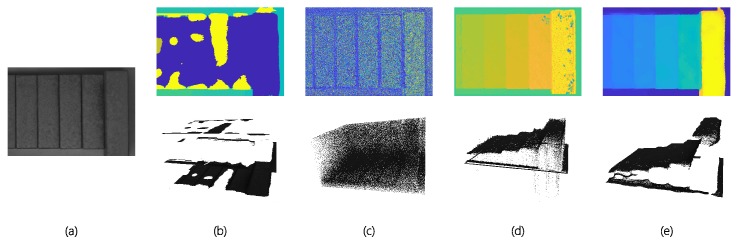
Standard gauge block results. The upper row is depth map and the lower is 3D point cloud. (**a**) thumbnail; (**b**) [6]; (**c**) [8]; (**d**) [31]; (**e**) Ours(VommaNet).

To validate the accuracy of our measurement system, we test our system with 6 standard gauge blocks of 3, 4, 5, 6, 8 and 16 mm in height separately. When measuring, an industry grade monochrome light-field camera (VOMMA Optec, VA4300M) is used instead of Lytro Illum camera, because we cannot obtain accurate MLA parameters from Lytro cameras. The raw light-field image is taken by the camera viewing vertically through the coaxial light. Also, we follow the MLA calibration procedure described in [5] and the metric calibration procedure from [44]. The image is shown in Figure 5. To further demonstrate the performance of the neural network we propose, we conduct depth estimation using several state-of-the-art algorithms as well as our network with the same SAIs as input, and compare the results both qualitatively in depth image and quantitatively in measurement accuracy and runtime. All the results are calculated on the same workstation with Intel E5-2603 v4 @1.7GHz, 64GB RAM, and Nvidia GeForce GTX 1080Ti, therefore, we can measure the runtime for each algorithm. When measuring the accuracy of different algorithms, since the heights of all the gauge blocks are known, we can calculate the heights of the blocks from the measurement results of different algorithms, and then obtain the accuracy of each algorithm by compare the measured heights with the ground truth heights. The results are shown in Figure 6 and Table 2.

From Figure 6b we can see that, since the metal-made blocks are reflective and texture-less, algorithm from [6] cannot yield correct results. The same is true for algorithm from [8], which can be seen from Figure 6c. We think this is because these two algorithms are based on EPI, and since the metal blocks are reflective and texture-less, it’s hard to find the correct EPI slope value for them, as a result, the algorithms will yield absurd results. Therefore, the average error and standard deviation values for these two algorithms are pretty large. Also, although result of the algorithm from [31] is pretty good for most of the blocks, it still gives absurd values for the two blocks on the right due to texture-lessness, which results in large maximum error and average error values. We think this is because this algorithm uses neural network to extract EPI slopes and then estimate depth from them, therefore, in regions where the effects of reflection or texture-lessness are subtle, it can calculate relatively accurate results, however, if the effects become prominent, the algorithm will fail and yield absurd value. In comparison, our algorithm yield a better result. Although there are still some outlier points and less accurate regions, the overall average error value and standard deviation value are very small, which can demonstrate the accuracy of our algorithm.

## 5. Conclusions

In this study, we propose a fast and accurate 3D measurement system based on a single light-field camera and our newly proposed light-field depth estimation neural network. As demonstrated previously, our network has good performance in reflective and texture-less areas as well as ordinary ones. Meanwhile, our network has achieved better overall accuracy than existing methods while maintaining similar runtime. Meanwhile, our measurement system can measure the 3D geometry of objects with one single shot from its light-field camera, and achieves an accuracy of 0.31 mm within 0.52 s.

Although our network performs well in reflective and texture-less areas, it does bad in preserve details. Complicated structures within objects may be blurry in our network, while texture from background or on object surfaces may be preserved to some extent. Also, our network is trained to take a specific number of SAIs as input, and this can be improved by modify the network to be recurrent, similar to [45]. This way, we will be able to train one single model for different numbers of input images, further extending the application of our network. Besides, the measurement accuracy of our system can be further improved by replacing the monochrome light-field camera we currently use by a color one as well as by developments in high-resolution light-field cameras.

## Figures and Tables

**Table 1:** Results comparison. Runtime is reported by author. For both scores, lower is better.

Method	MSE $\times 10^{-2}$	Runtime/s
[8]	3.968	2115.407
[31]	2.521	2.041
Ours(VommaNet)	2.151	2.043

**Table 2:** Results comparison on gauge block measurements with different depth algorithms.

Method	Avg. Error/mm	Std/mm	Max Error/mm	Min Error/mm	Runtime/s
[6]	10.6110	4.2124	18.5052	0.6887	316.527
[8]	6.3641	4.3142	18.8065	0.0200	481.407
[31]	1.2407	2.5277	40.5208	0.0000	0.841
Ours	0.3059	0.3408	7.9003	0.0000	0.543
